# Alcohol use and health outcomes in the oldest old

**DOI:** 10.1186/1747-597X-1-8

**Published:** 2006-03-29

**Authors:** Carolyn L Turvey, Susan K Schultz, Dawn M Klein

**Affiliations:** 1 Department of Psychiatry, University of Iowa, Iowa City, IA, USA

## Abstract

**Background:**

As the United States population ages, an unprecedented proportion of the population will be aged 70 and older. Knowledge of alcohol use and its consequences in this age group is not well known. In light of the disparate findings pointing to negative outcomes with excessive drinking yet also benefits of moderate drinking, the true risk of alcohol use in late life needs more investigation.

**Methods:**

This study examined the correlates and 2-year health outcomes related to alcohol use in 7,434 elders aged 70 years or older. Data was collected as part of the Assets and Health Dynamics of the Oldest Old (AHEAD), a nationwide health and economic study of elders. Data from Wave 1 and Wave 2 of AHEAD are presented. Frequency and quantity of drinking was assessed by self-report as was health status, lifetime alcohol or psychiatric problems, presence of chronic illness, functional impairment, and depressive symptoms. Cognitive status was assessed using a brief measure.

**Results:**

Approximately 44% of the sample reported any alcohol use in the past three months, with the majority of drinking less than daily. Daily drinking was associated with being Caucasian, married, in relatively good health, and having good affective and cognitive status. Drinking was not associated with negative health outcomes two years later and was protective against stroke and functional impairment. Decline in drinking between Wave 1 and Wave 2 was strongly associated with poor health.

**Conclusion:**

This study offers no evidence of negative health outcomes for drinking moderately and confirms the U-shaped curve often found in studies of alcohol and health. Nonetheless, cessation of drinking was associated with poor health suggesting the health benefits of moderate drinking may result from selection of a healthy group of people capable of sustained moderate drinking. Public health recommendations for moderate drinking must take this phenomenon into account.

## Background

The age structure of the US population is making an unprecedented shift towards growing proportions aged 65 and older. The number of Americans aged 65 and older grew from 3.1 million in 1900 to 35.3 million in 2001[1]. This corresponded to a growth from 4% of the population in 1990 to 12.4% in 2001. With the aging of the baby boomers, this is expected to rise to 70.3 million by 2030, about 20% of the total projected US population[2]. The highest rate of increase within those aged 65 and older is in the oldest-old, those aged 85 and older. This age group is expected to almost double between now and 2030, growing from 4.4 million to 8.9 million[2].

The aging of the US population calls for more research on the prevalence, risk factors and consequences of alcohol use in late-life. In contrast to the vast literature on alcohol use and abuse in younger age groups, relatively little is known about drinking patterns in the elderly, particularly the oldest-old. Of the two largest community based studies of psychiatric disorders, the Epidemiologic Catchment Area Study (ECA) and the National Comorbidity Survey, only the former included people aged 65 and older. The ECA estimates alcohol abuse in this age group to range from 1.9 to 4.6% for men and from 0.1% to 0.7% for women[3]. This study was conducted in the early 1980's. The National Household Survey on Drug Abuse was conducted more recently between 1991 and 1993[4]. It examined patterns of alcohol use as well as dependence. It estimated that 54.9% aged 50 and older used alcohol, but alcohol dependence was 1.6%, comparable to that found in the ECA. This sparse literature demonstrates that there is significant risk for alcohol abuse in late-life, though both studies found the risk to be much lower when compared to younger age groups.

Drinking in late-life has become a more complicated issue in light of the growing literature on the health benefits of moderate drinking and the relatively lower risk for alcohol abuse. Whereas abstinence was once considered the healthiest option, more and more physicians and public health leaders are following an informal clinical policy of recommending moderate drinking, usually defined as one drink per day. Several studies in predominantly middle aged samples (mean age between 50 and 60 years) have found a benefit of moderate drinking, particularly for cardiovascular outcomes [5-8]. Both men and women who drink approximately 1 drink per day have a lower relative risk of coronary disease when compared to non-drinkers or heavy drinkers. This "U-Shaped" curve is well established though speculations on the reasons underlying the curve are controversial.

Those that propose a direct benefit of alcohol cite the effect of raising high- density lipoprotein (HDL) cholesterol levels in moderate users [9,10]. Furthermore, cardioprotective benefits may be related to alcohol's ability to decrease platelet aggregation [11] and increase the prostacyclin/thromboxane ratio[12,13], resulting in a reduced propensity to thrombosis. Others argue that the U-Shaped curve is an artifact of the demographic group most likely to engage in moderate drinking [12-16]. Therefore, it is not the alcohol that leads to health benefits, but rather social and personality factors that can sustain long periods of controlled drinking without leading to excessive use. Moreover, many abstainers are people who previously used alcohol, but due to the development of health problems, had to stop drinking [15,16].

In support of this, the British Regional Heart Study of middle aged British men found that 70% of non-drinkers are ex-drinkers; thereby contaminating comparisons of the long-term health risks and benefits of alcohol use[15,16]. Similarly, Krahn et al [14] found the demonstrated benefits of moderate drinking were no longer evident after controlling for baseline cognitive ability and educational attainment. This study had the benefit of measures of cognitive ability in the late teens presumably prior to any extensive alcohol use. However, subsequent studies have controlled for prior alcohol use or education level and the moderate benefit for cardiovascular outcomes remains[5,17].

It is unclear if these benefits of moderate drinking hold in late-life. A separate examination of elders only is needed because of the issue of competing risks, particularly in more vulnerable elderly. Taking into account competing risks means examining the full risk profile for a certain behavior rather than examining its impact on one outcome. Although cardiovascular health is enormously important in aging, the recommendation of moderate alcohol use must take into account alcohol's potential to cause other negative outcomes in late-life. The benefits of moderate drinking need to be weighed in light of the risk they pose to other key geriatric syndromes such as cognitive impairment, depression, falls, and hip fracture.

The negative effects of alcohol use relate mostly to excessive drinking. Alcohol abuse can lead to pancreatitis, cirrhosis, or alcohol-related cardiomyopathy[18]. It may also lead to impaired driving, falls, memory problems, depression, and sleep problems [19-21]. It is unclear whether moderate drinking in late-life increases risk for these outcomes. Looking at a range of health outcomes will demonstrate whether the benefit of moderate alcohol use increases other health risks. It will also test whether the benefit of alcohol use is specific to cardiovascular outcomes or instead confers an overall protective effect. If the later is true, there may be broader underlying correlates of moderate alcohol use that account for the positive health outcomes.

This study presents data collected as part of a nationwide community-based survey of elders aged 70 and older who participated in the Assets and Health Dynamics of the Oldest Old (AHEAD)[22]. It has three aims. First, it will present data on drinking patterns in the oldest old. It will then present correlates of drinking in late-life. Finally, it will present longitudinal data on the short-term consequences of drinking in late-life. We will determine the relation between drinking and a wide range of health outcomes to examine whether the benefits of alcohol use are specific to cardiovascular outcomes or whether they are associated with overall health. The goal of this study is to inform clinicians and public health policy makers of the benefits and risks of alcohol use in late-life.

## Methods

AHEAD is a companion study to the Health and Retirement Survey and is intended to investigate the impact of health transitions on personal financial management, service and public program utilization, and intergenerational transfer of assets[22,23]. Wave 1 of AHEAD occurred in 1993–1994 and Wave 2 occurred in 1995–1996. The two sampling frames for the study were the 1992 screening of housing units enumerated for the Health and Retirement Survey and the Health Care Finance Administration's Master Enrollment file of Medicare enrollees who were living in a household. Primary respondents had to be 70 years or older and, if married, their partners participated regardless of their partner's age. Although the initial sampling frame excluded institutionalized elders, respondents who were institutionalized after Wave 1 remained in the study and were interviewed at Wave 2. All participants provided verbal informed consent and internal ethics review board approval was obtained.

The sample used in this study were all who participated in the first wave of AHEAD (N = 7,434) excluding spouses under the age of 70. Of these, 727 died between Wave1 and Wave 2. Of the living at Wave 2, 6,222 completed the second interview yielding a 93% follow-up rate. There is some variability in sample size for individual analyses due to missing data. Minor variability is due to non-response of participants and alters sample size by no more than ten observations. In addition, when proxy informants were used, the depression measure was not administered limiting the sample size to N = 6649 for analyses using this variable. Likewise, the cognitive measure was not administered when proxy interviews were done or the respondent was unable to complete the measure, limiting analyses including cognitive status to N = 6351. Demographic characteristics of the entire sample are provided in the second column of Table 2.

**Table 1:** Alcohol Consumption in Wave 1, percentage endorsing Cage and LifetimePsychiatric Problem and Alcohol Consumption in Wave 2. N = 7,434

Daily Alcohol Consumption Group	Daily Alcohol Use Wave 1- % (n) 7,434	% Wave 1 Consumption Group Endorsed Cage Item	% Wave 1 Consumption Group Endorsed Lifetime Psychiatric Problem	Daily Alcohol Use Wave 2 % (n) 6,184
None- Doesn't Drink	55.8 (4,146)	11.8 (491)	11.5 (478)	63.1 (3,903)
Less than once a day	33.8 (2,515)	11.5 (289)	9.8 (246)	27.3 (1,687)
1 to 2 drinks per day	8.3 (620)	22.3 (138)	9.7 (60)	8.0 (494)
3 or more drinks per day	2.1 (153)	49.0 (75)	15.7 (24)	1.6 (100)

**Table 2:** Demographic Correlates of Alcohol Consumption

Daily Alcohol Consumption	Total Sample N = 7,434	Doesn't Drink N = 4,146	Less than once a day N = 2,515	1–2 drinks per day N = 620	3 or more drinks per day N = 153	Test χ^2 ^statistic (df)	P <
% Male	39%	32.2	42.9	59.2	77.1	χ^2^(3) = 296.2	0.0001
Mean Age	77.6 (5.9)	78.3 (6.0)	77.0 (5.7)	76.5 (5.4)	74.5 (4.0)	χ^2^(3) = 157.3	0.0001
Race						χ^2^(6) = 182.6	0.0001
Caucasian	84.2	79.2	90.4	92.3	83.7		
African-American	13.8	18.2	8.3	6.3	13.7		
Asian/Pacific Islander/Other	2.1	2.6	1.3	1.4	2.6		
% Married	50.7	45.5	55.4	63.4	64.7	χ^2^(3) = 118.7	0.0001
Mean Education Yrs.	10.7 (3.8)	9.8 (3.9)	11.6 (3.4)	12.7 (3.2)	11.2 (3.4)	χ^2^(3) = 573.6	0.0001

Note: Kruskal Wallis tests were used for continuous variables (age, education) as these did not have normal distributions. Means and standard deviations are given for descriptive purposes only.

### Alcohol use and health measures

All health assessments were based on self report, except for cognitive status, which was based on the Telephone Interview of Cognitive Status-Revised[24]. Mortality data was collected in interviews of nearest kin and from the National Death Index.

#### Alcohol use

In Waves I and II, all participants were asked about frequency and quantity of drinking in the three months prior to the interview. Two questions assessed quantity of drinking "Do you ever drink any alcoholic beverages such as beer, wine, or liquor?" which was followed by "In general, do you have less than one drink a day, one or two drinks a day, three or four drinks a day, or five or more drinks a day." Participants were also asked the first item from the CAGE [25]screen for alcohol abuse: "At any time in your life, have you ever felt you should cut down on drinking?".

#### Health variables

These too, were based on self-report. Participants were asked to rate their health as excellent, very good, good, fair or poor. Participants reported whether a medical doctor had diagnosed them with cancer, heart disease, diabetes mellitus, high blood pressure, lung disease, stroke, or arthritis. They also reported whether they were current, former, or never smokers.

#### Falls and hip fracture

Participants were asked "Have you fallen down in the past 12 months?" and "Have you ever fractured your hip?"

#### Functional impairment

Measure of participant's functioning on activities of daily living (ADL's) and instrumental activities of daily living (IADLs) were assessed. In the ADL assessment, participants reported whether they needed help walking, dressing, bathing, eating, getting into bed, and using the bathroom[26]. These items were selected based on the original instrument described by Katz et al.[27] and subsequent revisions by Kane and Kane[28] and Weiner et al.[29] The assessment of IADL's included meal preparation, grocery shopping, telephone use, taking medication, and managing money. Item selection was based on Fillenbaum's revision [30]of Lawton and Brody's [31]original measure of IADL's. Participants were coded categorically as having any versus no difficulty in ADL's and IADL's because a large majority of participants reported no difficulty in either.

#### Neuropsychiatric variables

The AHEAD cognitive measure was adapted from the Telephone Interview for Cognitive Status[24], which was modeled after the Mini-Mental State Examination [32]to be administered over the telephone. A total score (range 0 – 35) was determined by summing the serial 7, immediate and delayed free-recall, and the mental state scale totals. Depressive symptoms were assessed using an abbreviated 8-item version of Radloff's Center for Epidemiologic Studies Depression Scale[33]. The abbreviated version demonstrated a comparable factor structure and internal consistency to the 20 item version[34]. In addition, all participants were asked "Have you ever seen a doctor for emotional, nervous, or psychiatric problems?"

### Statistical analysis

All statistical analyses were conducted on SAS statistical software. Descriptive analyses were conducted using chi-square tests for categorical variables and a Wilcoxon rank-sum test or the Kruskal-Wallis Test for continuous variables. The longitudinal analysis used logistic regression for categorical variables and a general linear model for continuous variables. Two sets of longitudinal analyses were conducted which examined the impact of Wave 1 drinking on health outcomes at Wave 2 occurring two years later. The first set controlled only for the Wave 1 value for the predicted Wave 2 outcome. The second set controlled for age, sex and education and the Wave 1 value for the predicted Wave 2 outcome. Because the results were largely similar, only the latter are presented, and the former are available upon request.

Similarly, generalized estimating equations were conducted on the initial bivariate analyses presented in Tables 3 and 4. These analyses controlled for correlated observations due to sampling of participants' spouses or partners if they were married. The results of the generalized estimating equations and the simpler Wilcoxon or Kruskal-Wallis test were practically identical (analyses available upon request) indicating that the correlated observations did not alter the relation between alcohol use and health outcomes, thus we opted to conduct the simpler models and present these results. To control for multiple comparisons and large sample size, a significance level was set at p < 0.001. All tests were two-tailed.

**Table 3:** Health Correlates of Alcohol Consumption

Daily Alcohol Consumption	Total Sample N = 7,434	Doesn't Drink N = 4,146	Less than once a day N = 2,515	1–2 drinks per day N = 620	3 or more drinks per day N = 153	Test Statistic (df)	p-value
Self Rated Health Mean	3.1 (1.2)	3.3 (1.2)	2.8 (1.1)	2.6 (1.1)	2.6 (1.2)	χ^2^(3) = 383.9	0.0001
Mean # Chronic Illnesses	1.6 (1.2)	1.7 (1.2)	1.4 (1.1)	1.3 (1.1)	1.4 (1.2)	χ^2 ^(3) = 135.4	0.0001
% Stroke	10.6	12.6	8.4	7.4	5.9	χ^2 ^(3) = 41.2	0.0001
% Heart Disease	31.6	34.2	29.1	25.8	22.9	χ^2 ^(3)= 35.3	0.0001
% Diabetes	13.3	16.6	10.1	6.1	7.2	χ^2 ^(3) = 95.4	0.0001
BMI	25.4(4.5)	25.4(4.7)	25.5(4.3)	24.8(3.7)	25.5(3.8)	χ^2 ^(3) = 10.6	0.01
Smoking Status						χ^2 ^(6) = 452.7	0.0001
1 = Current	10.0	8.2	9.8	17.1	30.1		
2 = Former	42.5	35.5	48.3	61.3	60.1		
3 = Never	47.5	56.3	41.9	21.6	9.8		
% Falls	7.7	8.3	7.0	7.4	5.9	χ^2 ^(3) = 4.8	0.19.
% Hip Fracture	5.0	5.8	4.2	3.7	2.0	χ^2 ^(3) = 14.5	0.002
% any ADL	30.5	37.5	23.3	15.3	18.3	χ^2 ^(3) = 236.3	0.0001
% any IADL	31.0	38.5	22.4	19.5	15.0	χ^2 ^(3) = 253.1	0.0001
Mean CES-D*	1.7(1.9)	1.9(2.1)	1.5(1.9)	1.2(1.7)	1.3(1.7)	χ^2 ^(3) = 118.5	0.0001
Mean TICS-R*	19.5(5.9)	18.2(6.1)	20.8 (5.3)	21.6(4.6)	21.3(5.4)	χ^2 ^(3) = 366.0	0.0001

Note: Kruskal Wallis tests were used for continuous variables (BMI, TICS-R) as these did not have normal distributions. Means and standard deviations are given for descriptive purposes only. BMI = Body Mass Index, ADL = Activities of Daily Living, IADL = Instrumental Activities of Daily Living, CES-D = Center for Epidemiologic Studies Depression Scale, TICS-R = Telephone Interview for Cognitive Status- Revised. *For analyses using the CES-D, N = 6,649. For those using the TICS-R, N = 6,351.

**Table 4:** Longitudinal Analysis: Wave 1 Alcohol Consumption predicting Wave 2 Health Outcomes. N = 6,222

	Estimate	Standard Error	Chi-Square (df = 1) or t-value	Odds Ratio	95% Confidence Interval	p-value
W1 drinking predicting stroke	-0.31	0.10	10.8	0.73	0.61–0.88	0.001
W1 drinking predicting heart disease	-0.2	0.08	5.8	0.82	0.69–0.96	0.02
W1 drinking predicting diabetes	-0.30	0.13	5.4	0.74	0.57–0.95	0.02
W1 drinking predicting falls	-0.09	0.06	2.5	0.91	0.81–1.02	0.12
W1 drinking predicting hip fracture	-0.42	0.17	5.8	0.66	0.47–0.92	0.02
W1 Drinking predicting % any ADL	-0.22	0.05	19.9	0.80	0.73–0.88	0.0001
W1 Drinking predicting % any IADL	-0.18	0.04	17.3	0.83	0.77–0.91	0.0001
W1 drinking predicting total # Chronic Illnesses	-0.03	0.01	-2.6	---	---	0.01
W1 drinking predicting CES-D	-0.1	0.03	-3.0	---	---	0.01
W1 drinking predicting TICS-R	0.17	0.08	2.14	---	---	0.03

Note: Each row represents the results for Wave 1 drinking from a separate multivariate model. Each model contained Wave 1 drinking consumption, age, sex, education and the Wave 1 value for the specific outcome (e.g. Wave 1 Tics-R score for the model predicting Wave 2 cognitive function). BMI = Body Mass Index, ADL = Activities of Daily Living, IADL = Instrumental Activities of Daily Living, CES-D = Center for Epidemiologic Studies Depression Scale, TICS-R = Telephone Interview for Cognitive Status- Revised. Due to missing data for proxy interviews, the n for analyses using the CES-D = 5,297 and for analyses using the TICS-R, the n = 5118.

## Results

Table 1 displays frequency and quantity of drinking in Wave 1 and Wave 2. In Wave 1, a little over 55% of the sample reported no drinking. Of the drinkers, the vast majority did not drink daily, while 8% drank one to two drinks per day. Two percent reported drinking three or more drinks per day. In Wave 2, occurring two years later, there was a general shift towards less drinking. In that wave, 63% reported no drinking while approximately 1.5% drank three or more drinks daily.

Table 1 also presents the percentage within each drinking consumption group reporting ever in their lifetime needing to cut back on drinking. The proportion endorsing this item gradually increased with each level of alcohol consumption, from 12% in the non-drinkers to 50% in those drinking three or more drinks per day. The percentage endorsing ever having had psychiatric problems in their lifetime follows the U or J-shaped curve with the non-drinkers and the 3-or-more drinks/day having the highest rates (11.5% and 15.7% respectively) and the moderate drinkers having the lowest rates at 9.7%.

The demographic correlates of drinking are provided in Table 2. Both gender and age showed a linear relationship, with greater drinking being associated with younger age and being male. Marriage was positively associated with moderate drinking. The relation to race and education was a bit more complicated. Moderate drinking when compared to no drinking was associated with being white and more years of education. A U-shaped pattern was seen for race, where moderate drinking was associated with being Caucasian, and either no-drinking or drinking three-or-more drinks per day was associated with being a minority.

Most of the negative health outcomes examined were higher in the non-drinkers and lower in the moderate drinkers (Table 3). Diabetes mellitus was also higher in the non-drinker group than the moderate drinkers. Cognitive function was higher for moderate drinkers and depression was lower. In contrast, the percentage currently smoking increased with increase in quantity of drinking.

Table 4 presents the value of Wave 1 drinking in predicting negative health outcomes at Wave 2. Each row presents the predictive value of Wave 1 drinking for the Wave 2 outcomes while controlling for age, sex, education and the Wave 1 value for the specific outcome (e.g. Wave 1 stroke for the model predicting Wave 2 stroke). Even in the controlled models, drinking conferred a protective benefit for stroke, and ADL and IADL functioning. Protection from heart disease, diabetes, and hip fracture did not reach the a-priori threshold set for statistical significance. Drinking did not appear to be associated with falls. Similarly, Wave 1 drinking was not associated with poorer cognitive function at Wave 2, depressive symptoms, or total number of chronic illnesses.

Of note, 727 participants died between Wave 1 and Wave 2. Alcohol use was strongly associated with mortality (χ^2^(df = 3) = 38.5, p < 0.0001). Twelve percent of the non-drinkers died while the mortality rate for those drinking less than once a day, once to twice a day or three or more times a day were approximately the same ranging from 6.5% to 7.6%. Therefore, any health benefits presented in Table 5 could be underestimates, since mortality was higher in the non-drinkers.

**Table 5:** Wave 1 predictors of a decrease in drinking between Wave 1 and Wave 2.

	Drank same amount Wave 1 and Wave 2 N = 1817	Decreased amount between Wave 1 and Wave 2 N = 991	Test Statistic (df)	p-value
Wave 1 Variable				
% Male	52.1	54.9	χ^2^(1) = 2.1	0.15
Mean Age (sd)	76.2(5.2)	77.0(5.8)	χ^2^(1) = 7.4	0.007
Race			χ^2^(2) = 27.7	0.0001
Caucasian	93.2	87.4		
African American	5.9	10.7		
Other	0.8	1.9		
% Married/partner	61.2	53.9	χ^2^(1) = 14.4	0.0002
Education Years (sd)	12.3(3.1)	11.2(3.6)	χ^2^(1) = 60.9	0.0001
Self Rated Health mean (sd)	2.6(1.1)	2.9(1.1)	χ^2^(1) = 60.1	0.0001
Mean # Chronic Illnesses (sd)	1.3(1.1)	1.4(1.2)	χ^2^(1) = 20.4	0.0001
% Stroke	6.7	8.6	χ^2^(1) = 3.5	0.06
% Heart Disease	25.3	30.5	χ^2^(1) = 8.7	.0003
% Diabetes	6.8	11.8	χ^2^(1) = 20.7	0.0001
% Falls	6.4	7.3	χ^2^(1) = 0.70	0.40
% Hip Fracture	3.7	3.2	χ^2^(1) = 0.49	0.48
% any ADL	15.3	25.9	χ^2^(1) = 47.0	0.0001
% any IADL	16.3	25.1	χ^2^(1) = 32	0.0001
Mean CES-D (sd)	1.1(1.6)	1.6(1.9)	χ^2^(1) = 37.9	0.0001
Mean TICS-R (sd)	21.8(4.8)	20.1(5.4)	χ^2^(1) = 59.1	0.0001

Note: Kruskal Wallis tests were used for continuous variables (age, education) as these did not have normal distributions. Means and standard deviations are given for descriptive purposes only. BMI = Body Mass Index, ADL = Activities of Daily Living, IADL = Instrumental Activities of Daily Living, CES-D = Center for Epidemiologic Studies Depression Scale, TICS-R = Telephone Interview for Cognitive Status- Revised. Due to missing data for proxy interviews, the n for analyses using the CES-D is 1737 and 903 for the two groups. For analyses using the TICS-R, the n = 1693 and 878 for the two groups.

The Wave 1 predictors of a decrease in drinking between Wave 1 and Wave 2 mirror the correlates of alcohol use in Tables 2 and 3 (Table 5). People who cut back on their drinking tend to be single, of minority status, and less educated. They are in worse health as indicated by the greater presence of heart disease, diabetes, and functional impairment. They also have significantly lower cognitive function and higher levels of depressive symptoms.

## Discussion

In the oldest old, moderate drinking is associated with better health. However, it is also related to demographic factors that are strongly associated with health outcomes. The relative contribution of these two factors to the apparent health benefits of moderate alcohol use needs to be better understood.

In this study, a little over one-half of the sample reported no alcohol use in Wave 1. This grew to 63% of the sample in Wave 2. These percentages of non-drinkers are far higher than those presented for younger age groups[4,8]. Although this can reflect cohort differences, it is more likely to result from the cumulative impact of drinkers stopping use because of ill-health or general aging. Along these lines, with increasing age the likelihood of prescription medication use becomes greater, often involving medications that preclude or warn again concomitant alcohol use. The analysis of decline in drinking presented in Table 5 provides a snapshot of the process contributing to non-drinking and its association with poor health. Although it may be accelerated in late-life, it seems safe to assume that this process is occurring throughout the life course. Therefore, health comparisons between drinkers and non-drinkers without controlling for prior drinking status are highly confounded. Moreover, given the large socioeconomic factors contributing to drinking status, it is not clear if controlling for prior drinking status would be sufficient.

Nonetheless, in the controlled models presented in this study, alcohol use did not appear to be associated with any negative health outcomes including falls and hip fracture. Indeed, it was associated with some health benefits such as stroke and physical function. Drinking was also associated with lower mortality between Waves 1 and 2. There appeared to be the potential for benefit for other health outcomes such as heart disease, but controlling for demographic factors mitigated this association considerably.

The Krahn et al study[14] noted earlier is unique in that it examined the relation between drinking and health while controlling for demographic and cognitive variables assessed long before this process of selection due to health on drinking has started. More studies like this are needed and some of the major longitudinal studies of aging, such as the Harvard Grant study [35]or the Seattle Longitudinal Study[36] should have the requisite data to conduct such an analysis.

This study suffers several limitations. All health variables are based on self-report, including that of alcohol use, a behavior that is often underreported. Moreover the questions forced respondents to group their alcohol use into a-priori categories. Assessments of depressive symptoms and cognitive status used validated measures, yet are briefer versions than those used in more detailed studies. The longitudinal analyses are based on a two-year period which is relatively brief when compared to other studies demonstrating the long-term implications of moderate alcohol use. Drinking was associated with lower mortality, so it is likely the benefits of drinking at Wave 2 were underestimated because the most ill non-drinkers died and were not represented in the longitudinal analyses. Finally, the analyses presented did not correct for correlated observations, yet exploratory analyses described in the Methods section strongly suggested that the results would not be altered greatly by doing so.

More research is needed to elucidate the mechanism by which alcohol use may benefit health in late-life. In addition, substance use varies widely by cohort. As the "baby-boomers" age, cohort specific research should be done on this group because of their known greater use of both alcohol and drugs.

## Conclusion

The health benefits of drinking observed in this study are not specific to cardiovascular illness, but are present for a range of medical and functional outcomes. Although the strong associations between moderate drinking and health outcomes supports the prophylactic use of alcohol, there also appear to be non-specific cultural factors that are tied to socioeconomic status. Given that socioeconomic status is one of the strongest correlates of health outcomes, these non-specific cultural factors need to be understood better before making unrestricted recommendations for moderate alcohol use. It should also be noted that moderate drinkers in this study were persons over the age of 70 who were able to maintain only moderate drinking, which may reflect a lower propensity to addictive behaviors or substance abuse disorders within this group. This is supported by the lower rates of smoking, depression and other psychiatric problems among the moderate drinkers. An informal clinical policy in favor of moderate drinking, then, may be appropriate only for a group with low risk for addictive disorders (e.g., no family of personal history of psychiatric disorders or substance abuse), but may not be appropriate for the general population.

## Competing interests

The author(s) declare that they have no competing interests.

## Authors' contributions

CT conceived of the study, participated in its design and helped to draft the manuscript.

DK conceived of the study and helped to draft the manuscript.

SS participated in the design of the study and helped to draft the manuscript.

All authors read and approved the final manuscript.
